# EMPOWERING VULNERABLE GROUPS IN LATER LIFE: FOCUS ON THE ROLE OF ACTIVITIES, SOCIAL NETWORK, AND ROUND-THE-CLOCK CARE

**DOI:** 10.1093/geroni/igz038.2139

**Published:** 2019-11-08

**Authors:** An-Sofie Smetcoren, G A Rixt Zijlstra

**Affiliations:** 1 Vrije Universiteit Brussel, Brussels, Belgium; 2 Maastricht University, Care and Public Health Research Institute (CAPHRI), Department of Health Services Research, Maastricht, Netherlands

## Abstract

Europe has been challenged with an intense rise of aging populations facing for example multiple chronic health problems, functional limitations and social and psychological challenges. With increasing age people may become vulnerable, nevertheless, they can still report high levels of well-being despite their deficits. Older adults’ strengths and resources can balance negative experiences and increase positive well-being outcomes. These resources can be personal (e.g. have sufficient income) or stemming from the social environment of the older person (e.g. an involved social network). Hence, this symposium focusses on these strengths and resources and how they might (positively) affect the well-being of vulnerable groups ageing in place. The main objective of the symposium is to give insights into different aspects and strategies that can protect older adults against negative outcomes. Four different studies from Belgium will be presented: Sarah Dury starts with explaining the potential buffering predictor of leisure and civic activities, by uncovering the mechanisms underlying the relationship between multidimensional frailty and well-being. Lise Switsers examines if the absence of social and emotional loneliness can act as a buffer to maintain a good well-being for older adults at risk of frailty. An-Sofie Smetcoren examines how ‘living in solidarity’ in a co-housing project can contribute to ageing in place. Finally, Sylvia Hoens explores the experiences of the older care users and their informal caregivers with live-in migrant care workers and examines how this care can increase their well-being.

